# The role and prospects of extracellular vesicles in advanced drug and vaccine delivery

**DOI:** 10.3389/fimmu.2026.1781365

**Published:** 2026-04-13

**Authors:** Defa Huang, Haibin Shen, Qing Jin, Tao Chen, Yuhuan Xie, Dingyu Rao, Meijin Liu

**Affiliations:** 1 Laboratory Medicine, First Affiliated Hospital of Gannan Medical University, Ganzhou, China; 2 The First School of Clinical Medicine, Gannan Medical University, Ganzhou, China; 3 Department of Cardiothoracic Surgery, The First Affiliated Hospital of Gannan Medical University, Ganzhou, China; 4 Laboratory Medicine, People’s Hospital of Ganzhou Economic Development Zone, Ganzhou, China

**Keywords:** cancer immunotherapy, drug delivery, extracellular vesicles, mRNA vaccines, vaccine delivery

## Abstract

Extracellular vesicles (EVs) have emerged as natural nanoscale carriers with exceptional biocompatibility, target specificity, and drug-loading capabilities, positioning them as promising tools in the field of drug and vaccine delivery. This review provides a comprehensive overview of the current research landscape surrounding EV-based delivery systems, highlighting their applications in mRNA vaccines and cancer immunotherapy. By examining the biological properties of EVs, along with contemporary methods for their isolation, modification, and functionalization, the review contrasts EVs with traditional nanocarriers such as lipid nanoparticles, emphasizing both their unique advantages and the challenges they face. Furthermore, we discuss recent clinical developments and ongoing trials that underscore the translational potential of EVs. Ultimately, this article aims to elucidate the pivotal role of EVs as next-generation platforms for advanced drug and vaccine delivery, offering insights into future research directions and therapeutic innovations.

## 1 Introduction

EVs are nanoscale, lipid bilayer-enclosed structures secreted by virtually all cell types into the extracellular milieu. These vesicles encapsulate a diverse repertoire of bioactive molecules, including proteins, nucleic acids (such as mRNAs, microRNAs, and DNA fragments), lipids, and metabolites, reflecting the physiological or pathological state of their cells of origin. EVs are heterogeneous in size and biogenesis pathways, comprising subtypes such as exosomes (30–150 nm), microvesicles, and apoptotic bodies, each with distinct origins and molecular compositions (1, 2). The lipid bilayer membrane of EVs not only protects their cargo from enzymatic degradation but also facilitates targeted delivery and uptake by recipient cells, thereby mediating intercellular communication across various biological contexts (3, 4). This unique biological function underlies EVs’ involvement in diverse cellular processes, including proliferation, apoptosis, migration, immune modulation, and disease pathogenesis (1, 5).

Traditional drug and vaccine delivery systems, such as liposomes, polymeric nanoparticles, and viral vectors, have been extensively explored for therapeutic applications. However, these conventional platforms often face significant limitations, including poor targeting specificity, suboptimal biocompatibility, potential immunogenicity, and rapid clearance from circulation, which collectively hinder their clinical translation and efficacy (6, 7). For instance, synthetic nanocarriers may induce off-target effects or immune responses that limit their safety profile, while viral vectors raise concerns about insertional mutagenesis and immunogenicity. Moreover, the delivery of sensitive biomolecules such as nucleic acids (e.g., mRNA, siRNA) remains challenging due to their instability and difficulties in traversing biological barriers (8, 9). These challenges necessitate the development of novel delivery platforms that combine high efficiency, safety, and targeting capabilities.

In this context, EVs have emerged as promising natural nanocarriers for advanced drug and vaccine delivery owing to their intrinsic biological properties. Their endogenous origin confers excellent biocompatibility and low immunogenicity, minimizing adverse immune reactions upon administration (10, 11). The lipid bilayer membrane and surface proteins of EVs enable them to evade rapid clearance and facilitate homing to specific tissues or cells, enhancing targeting precision (12, 13). Furthermore, EVs inherently possess the machinery for cargo loading and intercellular transfer, which can be harnessed or engineered to encapsulate therapeutic agents, including small molecules, proteins, and nucleic acids, for delivery to recipient cells (5, 14). Notably, EVs have demonstrated the ability to cross complex biological barriers such as the blood-brain barrier, expanding their utility in treating central nervous system disorders and tumors (15, 16).Recent advances have leveraged EVs in various therapeutic modalities. For example, EV-based drug delivery systems have been developed for targeted cancer therapy, improving drug bioavailability and reducing systemic toxicity (17, 18). EVs derived from immune cells or tumor cells have been investigated as vaccine platforms to elicit robust and specific immune responses against infectious diseases and cancers, with the potential for personalized immunotherapy (19, 20). Additionally, EVs have been explored as carriers for nucleic acid therapies, including mRNA and siRNA, addressing the challenges of stability and targeted delivery inherent to these molecules (9, 14). The application of EVs extends to emerging fields such as COVID-19 diagnosis, treatment, and vaccine development, highlighting their versatility and clinical relevance (21, 22).Despite their promising attributes, several technical and translational challenges remain in the clinical application of EVs. These include standardized methods for EV isolation, purification, and large-scale production, ensuring consistent quality and functionality (1, 23). Efficient and controlled cargo loading techniques, surface modification for enhanced targeting, and comprehensive understanding of EV biodistribution and pharmacokinetics are critical areas requiring further investigation (11, 24). Moreover, regulatory considerations and safety assessments must be addressed to facilitate the transition from bench to bedside (7, 25).

This review aims to systematically summarize the latest research progress on the roles of EVs as advanced drug and vaccine delivery vehicles. We will analyze their inherent advantages, elucidate current technological challenges, and discuss future prospects for their application in precision medicine. By integrating insights from recent studies, this article seeks to provide a comprehensive understanding of EVs’ potential to revolutionize therapeutic delivery systems and contribute to the development of next-generation nanomedicines.

## 2 Biological characteristics and classification of EVs

### 2.1 Sources and classification of EVs

**Figure 1:**
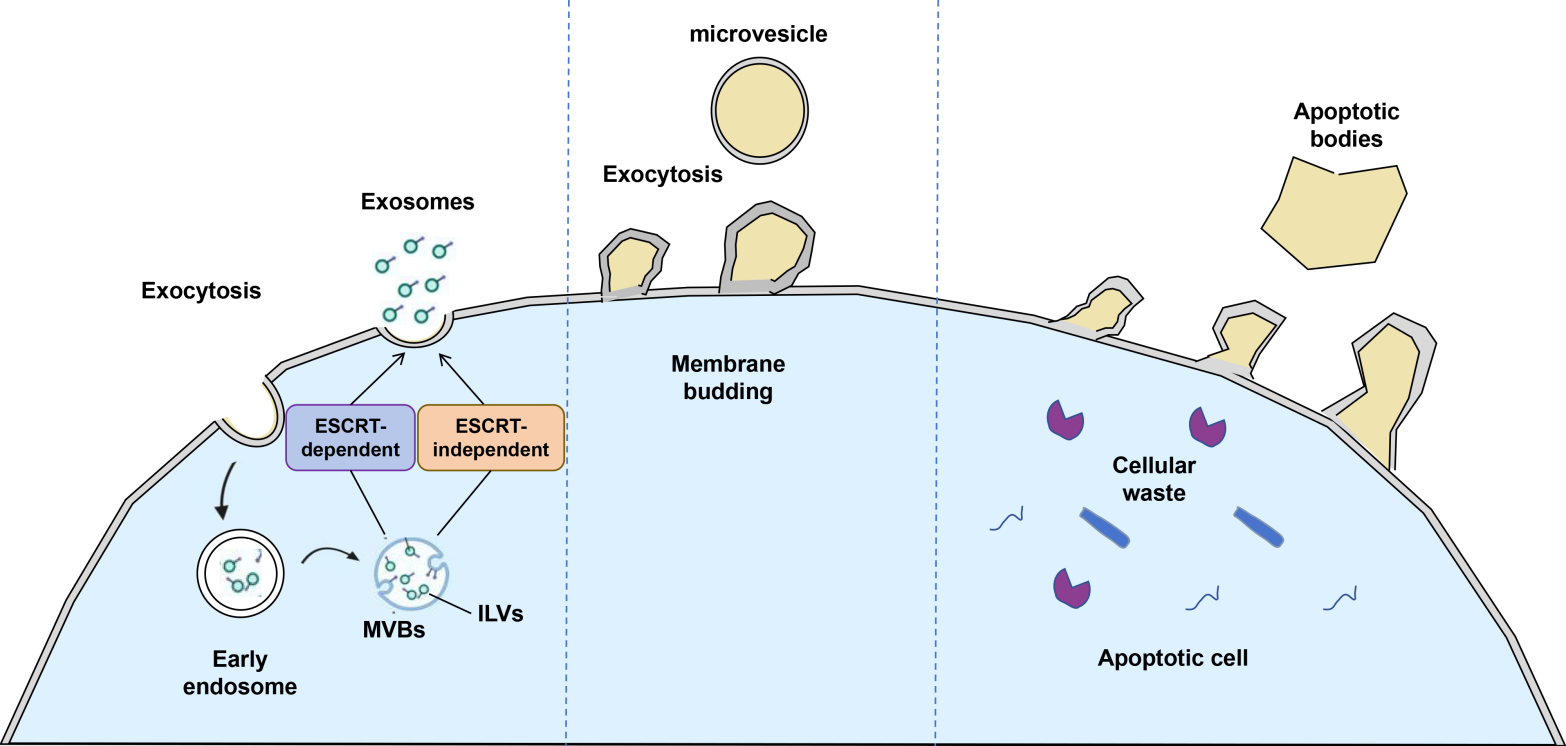
The biogenesis of extracellular vesicles involves several distinct mechanisms. Exosomes originate from the inward budding of the endosomal limiting membrane, leading to the formation of multivesicular endosomes, which are subsequently released into the extracellular space upon fusion of MVEs with the plasma membrane. In contrast, microvesicles are generated through outward budding and fission of the plasma membrane, a process often referred to as ectocytosis. These microvesicles, ranging in size from 100 to 1,000 nm, play a significant role in intercellular communication. Additionally, during the late stages of programmed cell death, apoptotic bodies are formed through membrane blebbing-a dynamic process involving extensive remodeling of the plasma membrane. Apoptotic bodies typically range from 50 to 5,000 nm in diameter and contain cellular debris and remnants of organelles. Their release facilitates the clearance of dying cells and contributes to immune regulation via antigen presentation.

EVs are a heterogeneous group of membrane-enclosed particles secreted by virtually all cell types, playing pivotal roles in intercellular communication by transporting bioactive molecules such as proteins, lipids, and nucleic acids. EVs are broadly classified into three main categories based on their biogenesis, size, and content: exosomes, microvesicles (also termed microparticles or ectosomes), and apoptotic bodies (**Figure 1**). Exosomes are small vesicles typically ranging from 30 to 150 nm in diameter, originating from the endosomal pathway through the inward budding of multivesicular bodies and subsequent fusion with the plasma membrane. Microvesicles are larger, ranging approximately from 100 to 1000 nm, formed by direct outward budding and fission of the plasma membrane. Apoptotic bodies are the largest EVs, generally between 500 and 1000 nm, released during the late stages of programmed cell death and often containing cellular organelles and nuclear fragments (26, 27). This size-based classification, while widely used, is complemented by molecular markers and cargo profiles to better delineate EV subtypes; for example, exosomes are enriched in tetraspanins such as CD9, CD63, and CD81, as well as proteins involved in endosomal sorting complexes required for transport (ESCRT) (28, 29).

The origin of EVs is crucial since vesicles derived from different cell types carry distinct protein and nucleic acid cargoes that influence their biological functions and targeting specificity. For instance, endothelial progenitor cell-derived EVs contain microRNAs and proteins that promote angiogenesis and exert anti-inflammatory effects in cardiovascular diseases, reflecting their parental cell phenotype (30). Similarly, EVs secreted by tumor cells harbor oncogenic proteins and nucleic acids that can modulate the tumor microenvironment and serve as biomarkers for cancer diagnosis and prognosis (31, 32). The molecular cargo of EVs is not only cell-type specific but also influenced by the physiological or pathological state of the originating cells, making EVs dynamic mediators of intercellular communication (33).Recent advances have highlighted the heterogeneity within EV populations even from a single cell type, attributed to differences in biogenesis pathways and cargo sorting mechanisms. Proteomic and lipidomic analyses have revealed distinct signatures between small and large EVs, suggesting functional divergence; for example, small EVs (exosomes) are enriched in proteins related to multivesicular body sorting, whereas large EVs (microvesicles) exhibit proteins associated with mitochondrial functions (34, 35). Moreover, the lipid composition of EVs varies with their size and origin, influencing membrane stability and interactions with recipient cells (29). The presence of specific lipids such as monoacyl glycerophosphate has been proposed as markers for endosomal origin, aiding in the classification of exosomes (29).Besides mammalian cells, EVs are secreted by a wide variety of organisms, including plants, bacteria, and parasites, broadening their biological relevance and therapeutic potential. Plant-derived nanovesicles have been explored as alternative sources for drug delivery due to their scalability and safety profile (36). Additionally, EVs from parasitic protozoa such as Leishmania carry virulence factors and RNA viruses that modulate host-pathogen interactions, emphasizing the diverse functional roles of EVs across species (37).

In summary, EVs encompass a spectrum of vesicles varying in size from approximately 30 to 1000 nm, including exosomes, microvesicles, and apoptotic bodies, each with distinct biogenetic origins and molecular cargoes. The specific protein and nucleic acid content of EVs reflects their cellular source and physiological context, determining their functional roles and targeting capabilities. Understanding the heterogeneity and classification of EVs is fundamental for harnessing their diagnostic and therapeutic potential in diverse medical fields (26, 29, 33, 34).

### 2.2 Biogenesis and release mechanisms of EVs

The biogenesis and release of EVs are complex processes involving multiple cellular pathways, primarily the endosomal multivesicular body (MVB) pathway and plasma membrane budding mechanisms. EVs encompass a heterogeneous population including exosomes, microvesicles, and apoptotic bodies, distinguished by their size, origin, and biogenesis routes. Exosomes are formed as intraluminal vesicles (ILVs) within MVBs, which are late endosomal compartments. MVBs can either fuse with lysosomes for degradation or with the plasma membrane to release their ILVs as exosomes into the extracellular space. This MVB pathway is tightly regulated by the Endosomal Sorting Complex Required for Transport (ESCRT) machinery, which orchestrates membrane budding and cargo sorting. For example, ESCRT components such as TSG101, Alix, and CHMP proteins participate in the formation of ILVs and sorting of specific cargo proteins, including viral oncoproteins like Epstein-Barr Virus LMP1, which hijacks this pathway to promote its secretion via EVs (38, 39). Additionally, ceramide, a sphingolipid, plays a crucial role in ESCRT-independent exosome biogenesis by inducing membrane curvature and budding, with ceramide transfer protein (CERT) mediating ceramide transport between the endoplasmic reticulum and endosomes, thereby regulating EV lipid composition and formation (40). Another important regulatory layer involves ubiquitination of adaptor proteins such as Arrdc4, which is essential for EV biogenesis and cargo trafficking, highlighting the role of specific post-translational modifications in vesicle formation (41).

Microvesicles, in contrast, are generated by direct outward budding and fission of the plasma membrane, a process influenced by cytoskeletal remodeling and intracellular calcium levels. Calcium influx triggers activation of enzymes such as calpain, which modulate cytoskeletal dynamics facilitating vesicle shedding. Studies comparing malignant and non-malignant cells reveal that malignant cells exhibit increased EV production, partly due to enhanced calcium signaling pathways, including store-operated calcium entry (SOCE), which sustains EV biogenesis after endoplasmic reticulum calcium store depletion (42). The release of microvesicles is spatially heterogeneous, occurring across the cell surface without preferred sites, and can be modulated by inflammatory stimuli such as TNF-α, which upregulates proteins like CD44 that are incorporated into EV membranes, influencing their biological effects and potentially contributing to disease progression such as atherosclerosis (43).

The release of EVs is highly regulated by the cellular state, microenvironment, and external stimuli. Mechanical forces, such as fluid shear stress, modulate EV secretion and alter their functional properties, exemplified by bone marrow-derived hematopoietic progenitor cells where different loading intensities affect EV release and their capacity to modulate osteoclast formation (44). Similarly, chronic contractile activity in skeletal muscle cells enhances EV secretion enriched with small EV markers, which mediate pro-metabolic effects in recipient myoblasts, indicating that physiological stimuli dynamically regulate EV biogenesis and cargo composition (45). In the context of immune and inflammatory responses, disturbed flow conditions in endothelial cells activate MAPK signaling pathways, increasing EV secretion that promotes inflammatory polarization of macrophages and accelerates atherosclerosis (46). Moreover, miRNAs serve as modulators of EV biogenesis and release; for instance, miR-125b-5p regulates the ESCRT-II complex via VPS36 in trophoblast cells, modulating EV subpopulation secretion during critical stages of implantation (47).Molecular machinery involving Rab GTPases, such as Rab27a/b and Rab11b, also governs EV trafficking and secretion. Rab27a/b are critical for the conventional exocytosis of EVs, as demonstrated in various cell types including ticks, where Rab27 silencing alters EV size distribution and impairs pathogen acquisition (48, 49). However, alternative pathways exist; during necroptosis, EV release can occur independently of Rab27, relying on lysosomal exocytosis, indicating flexibility in EV biogenesis mechanisms depending on cellular context (50). Additionally, autophagy-related pathways intersect with EV secretion, as secretory autophagy can mediate EV release when lysosomal degradation is impaired, highlighting crosstalk between degradation and secretion pathways (51).

In summary, EV biogenesis involves coordinated intracellular trafficking through the MVB pathway mediated by ESCRT complexes and ceramide-dependent mechanisms, as well as direct plasma membrane budding influenced by cytoskeletal and calcium signaling. The release process is dynamically regulated by cellular conditions, mechanical stimuli, inflammatory signals, and molecular regulators including miRNAs and Rab GTPases. Understanding these mechanisms provides insights into EV heterogeneity, cargo sorting, and their roles in physiological and pathological processes, paving the way for therapeutic and diagnostic applications leveraging EV biology (1, 52–54).

### 2.3 Biological activity and function of EVs

EVs are pivotal mediators of intercellular communication, exerting critical roles in physiological and pathological processes by transferring a diverse array of bioactive molecules, including proteins, lipids, nucleic acids, and metabolites, from donor to recipient cells. EVs are secreted by nearly all cell types and are involved in regulating recipient cell functions through delivery of their cargo, which can modulate gene expression, signaling pathways, and cellular phenotypes. This intercellular signaling capacity underpins their key roles in tissue repair, regeneration, immune modulation, and disease progression. For instance, EVs derived from endothelial or endothelial progenitor cells have been shown to induce cell proliferation and differentiation, inhibit apoptosis, and promote angiogenesis, highlighting their therapeutic potential in regenerative medicine (55). In immune regulation, platelet-derived EVs actively participate in inflammatory and autoimmune responses by mediating communication among immune cells and inflammatory factors. These EVs can transfer microRNAs to target cells, thereby modulating immune-inflammatory responses and offering novel therapeutic avenues for autoimmune diseases (56). Moreover, EVs carry microRNAs that are fundamental in regulating immunity, cancer progression, and inflammation, serving as crucial effectors in cell-to-cell communication (57). The cargo of EVs, including non-coding RNAs such as miRNAs, lncRNAs, and circRNAs, has been implicated in intracellular signal transduction and biological regulation, as exemplified by EVs derived from the anterior pituitary which may influence endocrine functions (58). Notably, EVs possess the inherent ability to penetrate biological barriers and stably transport bioactive molecules, which enhances their suitability as natural delivery vehicles. Their lipid bilayer membrane protects cargo from degradation, enabling stable transport in circulation and across tissue barriers, as demonstrated by EVs’ capacity to modulate hormone secretion and target distant cells (59). Furthermore, EVs exhibit high biocompatibility and low immunogenicity, allowing them to evade rapid clearance and immune detection, which is advantageous for therapeutic applications (60). Functionally, EVs influence various biological processes such as inflammation regulation, tissue regeneration, and immune modulation. For example, EVs derived from mesenchymal stem cells (MSCs) have shown anti-inflammatory and osteogenic activities, promoting tissue repair and regeneration in models of bone defects (61). In cardiovascular diseases, EVs secreted by diverse cell types modulate inflammation, angiogenesis, and cellular proliferation, contributing to both homeostasis and pathology (62, 63). EVs also mediate the transfer of functional molecules that regulate cellular metabolism and gene expression, as seen in microbial-derived EVs that carry small RNAs capable of cross-kingdom gene regulation (64). The surface proteins of EVs are crucial in mediating their interactions with recipient cells and the extracellular matrix, influencing uptake and functional outcomes (65). Moreover, EVs can be engineered or influenced by the external environment of parental cells to enhance their production and biological activity, which is a promising strategy to optimize their therapeutic potential (66). Taken together, the biological activity and function of EVs encompass a broad spectrum of intercellular signaling, immune regulation, and tissue remodeling processes, underpinned by their unique capacity to stably transport diverse bioactive molecules across biological barriers, making them indispensable players in health and disease as well as promising candidates for advanced drug and vaccine delivery systems.

## 3 Application of EVs in mRNA vaccine delivery

### 3.1 Comparison between EVs and lipid nanoparticles delivery systems

EVs and lipid nanoparticles (LNPs) are both lipid-based nanocarriers that have garnered significant attention for delivering nucleic acid therapeutics, including mRNA. Despite sharing a lipid bilayer structure, EVs are naturally secreted by cells and possess a complex composition of hundreds of lipid species, proteins, and carbohydrates, which confer unique biological properties such as enhanced biocompatibility, intrinsic targeting capabilities, and immune evasion (67, 68). In contrast, LNPs are synthetic constructs primarily composed of phospholipids, cholesterol, ionizable lipids, and PEGylated lipids, designed to encapsulate nucleic acids and facilitate cellular uptake and endosomal escape (69). A critical advantage of EVs over LNPs lies in their natural ability to protect and transport mRNA cargo with higher biological stability and reduced immunogenicity, which is essential for effective therapeutic delivery (70, 71). **Table 1** summarizes the comparison between EVs and LNPs in the delivery of advanced drugs and vaccines.

**Table 1:** Comparison of EVs and LNPs in advanced drug and vaccine delivery.

Comparison dimension	Extracellular vesicles (EVs)	Lipid nanoparticles (LNPs)
Source and nature	The naturally occurring nano-carriers are released by cells (such as immune cells, stem cells), and are used for intercellular communication.	A completely synthetic nano-carrier is composed of ionizable lipids, auxiliary phospholipids, cholesterol and PEG lipids, etc.
Component composition	The composition is complex, featuring a complex phospholipid bilayer and carrying various proteins (such as tetraspanins, integrins), nucleic acids and lipids from the source cell.	The composition is clear, with relatively simple and known components, usually precisely mixed from only 4 lipid components.
Drug loading	Challenges: The loading efficiency is relatively low, especially for large-molecule mRNA. Common methods such as electroporation or passive loading may cause damage to the cargo.	Advantages: Through microfluidic technology and the use of ionizable lipids for electrostatic interaction with nucleic acids, efficient encapsulation (>95%) can be achieved.
Targeting ability	Natural targeting: Through the surface proteins “barcodes”, it can achieve inherent tissue homing ability (such as crossing the blood-brain barrier).	Passive targeting is dominant: After intravenous injection, it mainly accumulates in the liver (through ApoE adsorption), and targeting tissues outside the liver requires complex modification.
Immunogenicity and safety	Low immunogenicity and high safety: As a natural substance, it has low immunogenicity and low cytotoxicity, making it more suitable for repeated administration or *in vivo* delivery.	Potential immune responses exist: Synthetic materials may trigger complement activation-related pseudo-allergic reactions, and polyethylene glycol components can induce the production of anti-polyethylene glycol antibodies.
Large-scale production	Major bottlenecks: The production process is complex, the output is low, separation and purification are difficult (such as removing shells and impurities), there are significant batch-to-batch variations, and the regulatory framework is not yet clear.	Mature process, easy to scale up: The GMP production process based on microfluidics or impinging jet mixing is mature, with good repeatability, and multiple products have been approved.

One of the most compelling distinctions between EVs and LNPs is their targeting efficiency, particularly to the lungs. Recent studies have demonstrated that lung-derived EVs (Lung-Exo) exhibit superior pulmonary targeting and retention compared to conventional LNPs when administered via inhalation. Unlike LNPs, which require extensive reformulation for inhaled delivery and often show limited pulmonary bioavailability due to rapid clearance and off-target distribution, Lung-Exo naturally home to bronchioles and lung parenchyma, facilitating enhanced mRNA translation and protein expression in pulmonary cells (72). This intrinsic lung tropism is likely attributed to the membrane proteins and lipid composition of Lung-Exo, which mimic the native pulmonary microenvironment and facilitate cellular uptake and endosomal escape more efficiently than synthetic LNPs (70, 73). Moreover, EVs possess superior endosomal escape mechanisms compared to LNPs, which is a critical barrier in nucleic acid delivery. While LNPs rely on ionizable lipids to destabilize endosomal membranes, their efficiency is often limited, leading to suboptimal cytosolic delivery of mRNA (70). EVs, by contrast, have evolved natural pathways to evade lysosomal degradation and promote cargo release into the cytoplasm, enhancing functional mRNA delivery and protein expression (70). This biological advantage translates into improved transfection efficiency and reduced cytotoxicity, as evidenced by hybrid systems combining EVs and LNPs that outperform conventional LNPs *in vitro* and *in vivo (*71, 74). In addition to biological advantages, EVs offer enhanced biocompatibility and reduced immunogenicity compared to LNPs, which can elicit unfavorable immune responses and toxicity due to their synthetic lipid components (75) (71). The natural origin of EVs allows them to evade immune clearance and prolong circulation time, although challenges remain in large-scale manufacturing and batch-to-batch consistency (67). Recent advances in microfluidic technologies have facilitated the scalable production of EV-LNP hybrid nanoparticles, combining the targeting and biocompatibility of EVs with the customizable payload capacity of LNPs, thereby addressing some of the translational hurdles (74).Furthermore, the lipid composition of EVs is more diverse and functionally complex than that of LNPs, including phosphatidylserine, phosphatidylcholine, cholesterol, and other bioactive lipids that contribute to membrane fluidity, stability, and cellular interactions (3, 76). This complexity enables EVs to interact with specific cell surface receptors, facilitating targeted delivery to tissues such as the lungs, which is difficult to replicate with synthetic LNP formulations.

In summary, EVs are naturally optimized for mRNA encapsulation and delivery, exhibiting higher biological stability, superior targeting-especially to pulmonary tissues-and enhanced endosomal escape capabilities compared to synthetic LNPs. Lung-derived EVs, in particular, demonstrate improved distribution and protein expression in the lung following inhalation, outperforming LNPs in preclinical models. However, challenges such as scalable production, cargo loading efficiency, and standardization remain for EVs, while LNPs benefit from well-established manufacturing processes but face limitations in biocompatibility and targeting specificity. Hybrid EV-LNP platforms and microfluidic production methods represent promising strategies to harness the advantages of both systems, potentially advancing the clinical translation of nucleic acid therapeutics (67, 68, 70, 72, 74).

### 3.2 Design and preparation technology of mRNA vaccines based on EVs vectors

The design and preparation of extracellular vesicle (EV)-based mRNA vaccines have advanced significantly through innovative loading techniques and stabilization strategies that address key challenges in mRNA delivery and vaccine distribution. One promising approach to achieve high-efficiency loading of mRNA or protein antigens into EVs involves the use of acoustic shock wave technology, exemplified by the Shock Wave Extracellular Vesicle Engineering Technology (SWEET). This acoustic shock wave-based post-loading method enables robust encapsulation of either mRNA or protein antigens into immunostimulatory EVs without compromising vesicle integrity. For instance, SARS-CoV-2 receptor-binding domain (RBD) protein and RBD mRNA were loaded into EVs derived from LPS-activated monocytes with encapsulation efficiencies of approximately 69% and 75%, respectively. The resulting EV vaccines elicited potent, adjuvant-free humoral and cellular immune responses in murine models, with neutralizing antibody titers and cytokine profiles comparable to or exceeding those induced by alum-adjuvanted controls. Notably, lyophilized EV vaccines retained immunogenicity after storage at 4 °C for seven days, demonstrating potential for cold chain-independent distribution (77).

Similarly, plant-derived EVs have been explored as natural carriers for mRNA vaccine delivery, leveraging their inherent stability and biocompatibility. EVs extracted from orange juice (oEVs) were efficiently loaded with SARS-CoV-2 mRNAs encoding various viral proteins, including the N protein and spike subunits. These oEVs protected the mRNA cargo from enzymatic degradation and harsh environmental conditions such as simulated gastric fluid, enabling oral and intranasal administration routes. Lyophilized oEVs maintained stability at room temperature for up to one year, facilitating vaccine storage and transport without the need for stringent cold chain logistics. Immunization of mice and rats via oral gavage or intranasal routes induced robust systemic and mucosal immune responses, including specific IgM, IgG, and secretory IgA production, as well as T cell activation. These findings underscore the feasibility of using edible plant-derived EVs for developing orally administrable mRNA vaccines with enhanced stability and immunogenicity (78–80).

Beyond natural EVs, hybrid EVs combining synthetic lipid nanoparticles (LNPs) and EV membranes have been engineered to improve mRNA loading efficiency and reduce cytotoxicity associated with synthetic carriers. These hybrid extracellular vesicles (HEVs) are formed by fusing EVs with LNPs under low pH conditions, preserving EV characteristics while incorporating LNP components. HEVs demonstrate superior transfection efficiency *in vitro* and *in vivo* compared to conventional LNPs, with significantly reduced cellular toxicity. Mechanistically, HEVs facilitate endosomal escape more effectively than native EVs, enhancing intracellular delivery of mRNA. *In vivo*, HEVs show preferential distribution to organs such as the spleen, suggesting potential for targeted vaccine delivery. This hybridization strategy represents a scalable and modular platform for mRNA vaccine development with improved safety and efficacy profiles (71, 81).

Another critical aspect of EV-based mRNA vaccine design is achieving room temperature stability to overcome cold chain limitations that have hindered widespread vaccine distribution, especially in resource-limited settings. Studies have demonstrated that lyophilized EV formulations, including those derived from lung tissue or plants, retain functional mRNA delivery capacity and immunogenicity after extended storage at ambient temperatures. For example, lung-derived EVs loaded with SARS-CoV-2 spike mRNA remained functional after one month of room temperature storage, outperforming synthetic liposomes in pulmonary delivery and immune activation upon inhalation. This stability enables the development of inhalable dry powder mRNA vaccines that are more accessible and easier to distribute globally (72, 82).

Collectively, these advances in EV-based mRNA vaccine design and preparation emphasize the integration of innovative loading techniques such as acoustic shock waves, hybridization with synthetic lipid carriers, and lyophilization for enhanced stability. These strategies enable efficient encapsulation of mRNA and protein antigens, protect cargo from enzymatic degradation, and facilitate diverse administration routes including oral, intranasal, inhalation, and parenteral delivery. The resultant EV vaccines exhibit strong immunogenicity, including mucosal and systemic immune responses, with reduced toxicity and improved storage profiles. Consequently, EV-based mRNA vaccines hold great promise for next-generation vaccine platforms that are safer, more effective, and more adaptable to global distribution challenges.

### 3.3 EVs-mediated mRNA vaccine immune activation mechanism

EVs have emerged as highly effective vehicles for delivering mRNA vaccines to antigen-presenting cells (APCs), thereby inducing potent humoral and cellular immune responses. EVs naturally encapsulate mRNA, protecting it from enzymatic degradation and facilitating its delivery to target cells, such as dendritic cells and macrophages, which are critical for initiating adaptive immunity. Several studies have demonstrated that EVs loaded with mRNA encoding viral antigens, such as the SARS-CoV-2 spike protein or its receptor-binding domain (RBD), can efficiently transfer these mRNAs into APCs, resulting in intracellular antigen expression and robust immune activation without the need for additional adjuvants. For example, EVs derived from lipopolysaccharide (LPS)-activated monocytes were post-loaded with SARS-CoV-2 RBD mRNA using acoustic shock wave technology, achieving high encapsulation efficiency (~75%) and eliciting strong neutralizing antibody titers alongside balanced Th1/Th2 cellular responses in mice, comparable or superior to alum-adjuvanted controls (77). Similarly, plant-derived EVs extracted from orange juice have been successfully loaded with SARS-CoV-2 mRNAs and administered via oral, intranasal, and intramuscular routes, inducing both systemic IgG and mucosal IgA responses, as well as T cell activation evidenced by IFN-γ production (78–80). These findings highlight the versatility of EVs in delivering mRNA vaccines through multiple administration routes, including non-invasive mucosal pathways, which are particularly advantageous for enhancing mucosal immunity and reducing cold chain dependency.

Beyond efficient mRNA delivery, EVs also possess intrinsic immunomodulatory properties that contribute to the activation of balanced Th1 and Th2 immune responses. This balanced activation is crucial for effective vaccine-induced protection, as Th1 responses promote cytotoxic T lymphocyte activity and cellular immunity, while Th2 responses support robust antibody production. EV-based mRNA vaccines have been shown to induce such balanced immunity, which is essential for durable and comprehensive protection against viral infections. For instance, engineered EVs carrying both mRNA and protein antigens of SARS-CoV-2 elicited potent neutralizing antibodies and antigen-specific T cell responses in preclinical models, demonstrating their capacity to integrate antigen delivery and immune stimulation within a single platform (83). Moreover, studies on EVs derived from bacterial outer membrane vesicles decorated with viral antigens have shown that intranasal immunization can induce high titers of systemic IgG and mucosal antibodies, along with neutralizing activity against SARS-CoV-2 variants, further underscoring the potential of EVs to stimulate both arms of adaptive immunity (84, 85).The ability of EVs to activate a balanced Th1/Th2 immune response also enhances vaccine efficacy by promoting the generation of mucosal IgA antibodies, which serve as the first line of defense at mucosal surfaces. Oral and intranasal administration of mRNA-loaded plant-derived EVs has been demonstrated to induce specific IgA production, contributing to mucosal barrier immunity and potentially reducing viral transmission (78–80). This mucosal immunogenicity is a significant advantage over conventional lipid nanoparticle-based mRNA vaccines, which predominantly induce systemic immunity and limited mucosal responses (86). Furthermore, EVs’ capacity to maintain mRNA stability at room temperature after lyophilization supports their practical application in vaccine distribution, especially in resource-limited settings (77, 79).At the molecular level, EV-mediated mRNA vaccine delivery leads to the internalization of mRNA by APCs, followed by antigen translation and presentation via major histocompatibility complex (MHC) molecules, thereby activating CD4+ helper and CD8+ cytotoxic T cells. This process is accompanied by the secretion of proinflammatory cytokines and chemokines, which further enhance immune cell recruitment and activation. For example, EVs carrying SARS-CoV-2 antigens have been shown to activate MAPK signaling pathways and upregulate chemokines such as CXCL-10 and CXCL-11, as well as cytokines like IL-6, indicating a robust innate immune activation that supports adaptive responses (87). Additionally, circulating EV microRNAs have been identified as biomarkers correlating with vaccine-induced antibody production and inflammatory responses, suggesting that EV cargo modulates immune regulation post-vaccination (88, 89).

In summary, EVs serve as efficient and versatile carriers for mRNA vaccines, facilitating targeted delivery to APCs and inducing strong humoral and cellular immunity. Their intrinsic ability to activate balanced Th1/Th2 responses enhances vaccine efficacy by promoting both systemic and mucosal immunity. The stability, biocompatibility, and immunostimulatory properties of EVs position them as promising next-generation platforms for mRNA vaccine delivery, with potential applications extending from infectious diseases to cancer immunotherapy. Continued advancements in EV engineering and loading technologies are expected to further optimize their immunogenicity and clinical translation prospects.

## 4 The role of EVs in tumor drug delivery and immunotherapy

### 4.1 Advantages of tumor cell-derived EVs in drug delivery

**Figure 2:**
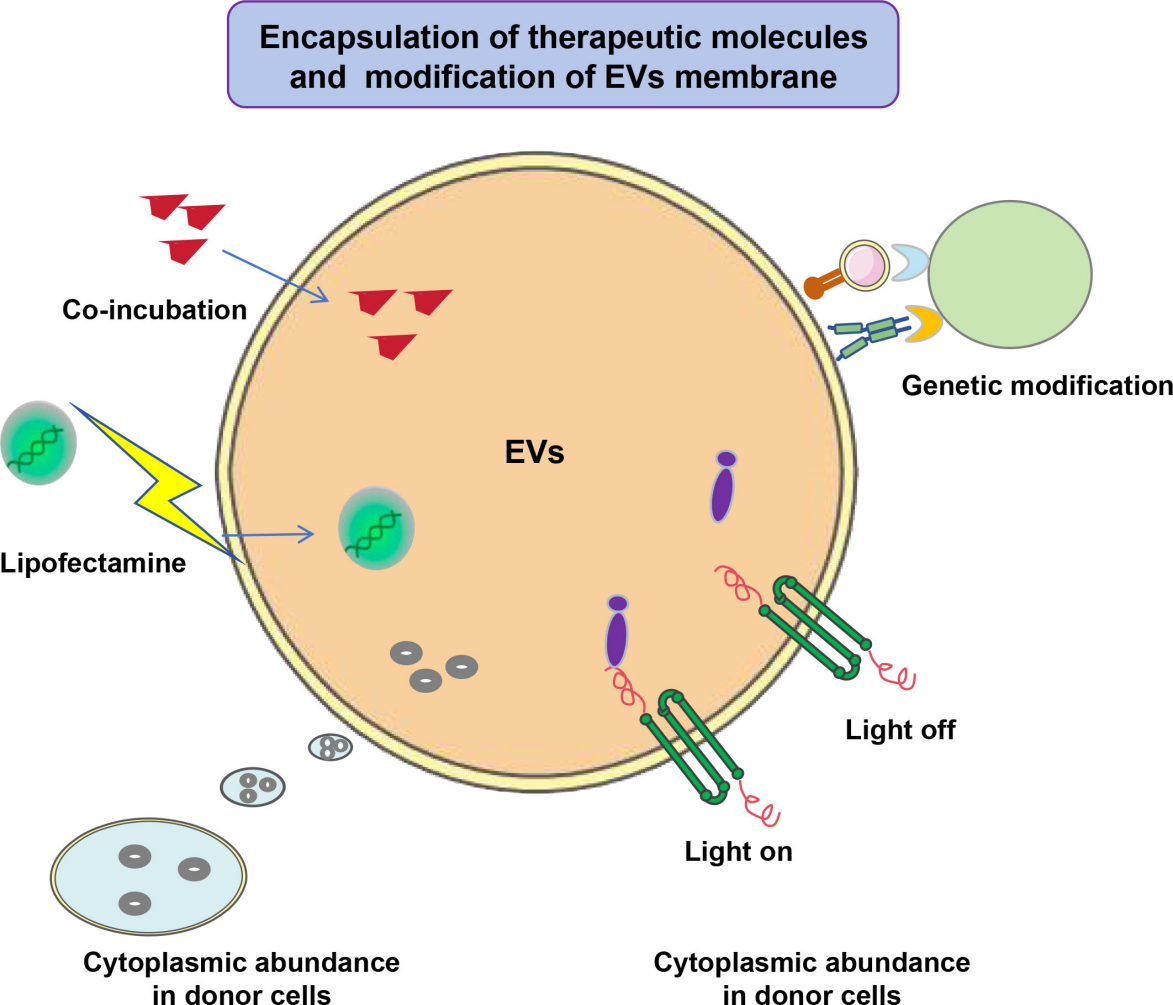
Engineering methods of EVs as a nanoplatform for improving therapeutic efficacy. EVs could be therapeutically engineered with the encapsulation of therapeutic molecules and modification of the EVs membrane. Encapsulation methods involve co-incubation (direct mixing), membrane permeability enhancement (physical/chemical stimuli), cytoplasmic abundance in donor cells, and selective encapsulation via machinery related with EVs biogenesis and release. Membrane modification methods involve genetic modification.

Tumor cell-derived extracellular vesicles (TEVs) possess distinct advantages in drug delivery, primarily due to their inherent compatibility and homing ability toward their parental tumor cells. This homotypic targeting arises from the presence of tumor-specific membrane proteins and lipids on TEVs, enabling them to selectively recognize and fuse with tumor cells of the same origin, thereby achieving precise drug localization and minimizing off-target effects. With the advancement of nanotechnology, nanomedicine has significantly enhanced the loading capacity, biodistribution, and targeted accumulation of therapeutic molecules. These advancements have been effectively applied to EVs through the encapsulation of therapeutic agents and the engineering of EV membranes (**Figure 2**). For instance, a novel nanoplatform integrating breast cancer cell membrane fragments with lemon-derived nanovesicles demonstrated enhanced homologous tumor targeting and efficient transcellular drug transport, resulting in significant tumor growth inhibition without observable toxicity upon intravenous administration (90). This exemplifies how embedding tumor cell membrane components onto vesicles can confer superior tumor specificity. Furthermore, TEVs can encapsulate a broad range of therapeutic agents, including chemotherapeutic drugs, small interfering RNAs (siRNAs), and immune modulators, thereby enhancing antitumor efficacy through multifaceted mechanisms. Their phospholipid bilayer structure provides a protective environment for these cargos, improving stability and bioavailability. Studies have shown that TEVs can deliver chemotherapeutics like doxorubicin and oxaliplatin effectively to tumor cells, with engineered modifications such as surface functionalization with targeting ligands (e.g., hyaluronic acid targeting CD44 in hepatocellular carcinoma) further augmenting delivery precision and therapeutic outcomes (91, 92). Additionally, TEVs loaded with RNA interference molecules have been used to overcome drug resistance in breast cancer by facilitating selective delivery and reducing off-target toxicity (93).

The unique ability of TEVs to modulate the tumor microenvironment also contributes to their therapeutic potential. By delivering immune regulatory molecules or inducing apoptosis in tumor cells, TEVs can disrupt tumor progression and sensitize tumors to chemotherapy. For example, TEVs have been shown to enter tumor stem cells and deliver drugs directly to the nucleus, effectively killing these cells while sparing normal tissues due to low immunogenicity and efficient tumor homing (94). Moreover, TEVs can be engineered to carry antibodies or ligands that target senescent tumor cells, thereby overcoming therapy-induced senescence and enhancing chemotherapy efficacy (95). Despite these promising features, challenges such as low drug-loading capacity, heterogeneity of vesicle populations, and potential safety concerns remain. Advances in engineering approaches, including surface modification, cargo loading techniques, and hybridization with synthetic nanoparticles, are being explored to overcome these limitations and improve therapeutic performance (96, 97). The integration of TEVs with other drug delivery platforms, such as mesoporous silica nanoparticles coated with tumor-derived EV membranes, has demonstrated enhanced selective intracellular drug accumulation and cytotoxicity against cancer cells (98).

In summary, TEVs offer a natural, biocompatible, and highly specific drug delivery system that can carry diverse therapeutic agents directly to tumor cells, thereby enhancing antitumor efficacy and reducing systemic toxicity. Their tumor-homing capabilities, combined with the ability to modulate the tumor microenvironment and deliver multifunctional cargos, position TEVs as a powerful platform for precision oncology. Ongoing research into engineering strategies and clinical translation will be critical to fully realize their potential in cancer therapeutics.

### 4.2 EVs combined with immune checkpoint inhibitors

The combination of extracellular vesicles (EVs) with immune checkpoint inhibitors (ICIs) represents a promising strategy to enhance the efficacy and safety of cancer immunotherapy. EVs, as natural nanoscale carriers, can be engineered to deliver antibodies targeting immune checkpoints such as PD-1/PD-L1 and CTLA-4, thereby improving the precision of immune modulation within the tumor microenvironment. By encapsulating these antibodies within EVs, it is possible to increase their stability, bioavailability, and tumor-targeting capacity, while potentially reducing systemic toxicity commonly associated with systemic ICI administration. For example, EVs loaded with ICIs have demonstrated improved delivery across biological barriers, such as the blood-brain barrier, enabling enhanced treatment of brain tumors like glioma (99). Moreover, EVs can be surface-engineered to target specific tissues or tumor-associated cells, further refining the immune checkpoint blockade to the tumor site and diminishing off-target effects (100, 101). This targeted delivery not only potentiates the antitumor immune response by reinvigorating exhausted T cells but also mitigates immune-related adverse events by limiting systemic exposure to checkpoint inhibitors. Preclinical animal studies have provided compelling evidence that combination therapy using EVs and ICIs can synergistically enhance antitumor immunity. In melanoma and lung cancer models, dendritic cell-derived EVs loaded with tumor antigens and combined with anti-PD-1 or anti-PD-L1 antibodies elicited robust antigen-specific T-cell responses, leading to significant tumor regression and prolonged survival (102). Similarly, EVs engineered to carry PD-L1 siRNA or antibodies have been shown to reverse immune suppression mediated by tumor-derived EV PD-L1, thereby restoring T cell function and sensitizing tumors to ICIs (103, 104). Furthermore, EVs enriched with antiangiogenic factors have been used to normalize tumor vasculature, enhancing immune cell infiltration and potentiating the efficacy of anti-PD-1 therapy in resistant tumor models (105).

Clinical investigations also suggest that circulating EVs expressing immune checkpoint molecules can serve as predictive biomarkers for ICI therapy response and toxicity. For instance, elevated levels of PD-L1-positive EVs correlate with poor response to ICIs and may contribute to immune evasion mechanisms (106, 107). Monitoring dynamic changes in EV PD-L1 during treatment can aid in distinguishing true progression from pseudoprogression, thus guiding clinical decision-making (108). Additionally, EV cargo such as TGF-β and specific microRNAs have been implicated in modulating the tumor immune microenvironment, influencing both therapeutic resistance and efficacy of ICIs (109, 110). These findings underscore the dual role of EVs as both therapeutic vehicles and biomarkers in immune checkpoint blockade strategies. Importantly, the EV-mediated delivery of ICIs has been associated with a reduction in immune-related adverse events. For example, redirecting tumor-derived EVs to non-tumor tissues like the heart has been demonstrated to alleviate ICI-induced cardiotoxicity while maintaining antitumor efficacy (111). This approach highlights the potential of EVs to improve the safety profile of ICIs by modulating immune checkpoint pathways in a tissue-specific manner.

In summary, the integration of EVs as delivery platforms for immune checkpoint inhibitors offers a multifaceted advantage: enhancing targeted delivery and therapeutic efficacy, reducing systemic toxicity, and providing novel biomarkers for treatment monitoring. Ongoing research continues to refine EV engineering techniques, optimize loading strategies, and evaluate combinatorial regimens in preclinical and clinical settings. These advances position EV-based combination therapies as a frontier in precision immunotherapy, with the potential to overcome current limitations of immune checkpoint blockade and improve outcomes for cancer patients.

### 4.3 EVs as cancer vaccine platforms

EVs have emerged as a highly promising platform for cancer vaccine development due to their intrinsic ability to carry tumor-associated antigens and stimulate specific T cell-mediated immune responses. Unlike traditional cancer vaccines, which often suffer from limited immunogenicity and suboptimal antigen presentation, EVs naturally encapsulate a broad spectrum of tumor antigens, including neoantigens, and present them in the context of major histocompatibility complex (MHC) molecules. This property enables EVs to effectively prime and activate antigen-specific CD8+ cytotoxic T lymphocytes (CTLs) and CD4+ helper T cells, overcoming the immunogenicity challenges faced by conventional vaccines (20, 112). EVs derived from dendritic cells (DC-EVs) are especially notable for their immunostimulatory cargo, including preformed peptide-MHC complexes and co-stimulatory molecules, which facilitate direct activation of T cells and enhance dendritic cell maturation, thereby amplifying antitumor immunity (113, 114). Furthermore, tumor-derived EVs (TDEVs) can be engineered or modulated to reduce their immunosuppressive components while enriching immunogenic molecules, as demonstrated by strategies such as inducing endoplasmic reticulum stress in tumor cells to produce immunogenic EVs with enhanced adjuvant properties (115). This approach not only activates dendritic cells but also promotes cytotoxic T lymphocyte responses that inhibit tumor growth and recurrence. Importantly, EVs can be loaded with adjuvants, such as toll-like receptor agonists, or combined with other immunomodulatory agents to further potentiate antitumor responses, as shown in preclinical models where EV vaccines conjugated with TLR9 agonists achieved significant tumor-free survival rates (112, 116). The versatility of EVs extends to their ability to carry chimeric RNA-encoded neoantigens, enabling the generation of personalized cancer vaccines that elicit potent CD8+ T cell-mediated immunity even in tumors with low mutational burden (117). Additionally, EVs derived from various immune cells, including macrophages and NK cells, can be engineered to enhance their antigen presentation and immune activation capabilities, broadening the scope of EV-based vaccine platforms (118). The inherent biocompatibility, low immunogenicity, and natural targeting properties of EVs confer advantages over synthetic nanocarriers, facilitating efficient delivery to lymphoid organs and tumor microenvironments (119, 120). Moreover, EV vaccines produced under Good Manufacturing Practice (GMP) standards offer high safety profiles, ease of storage, and transportability, addressing critical logistical challenges in vaccine deployment (20). Clinical trials with dendritic cell-derived EV vaccines have demonstrated manageable safety and some immunological efficacy, although challenges remain in optimizing immunogenicity and overcoming tumor-induced immunosuppression (121, 122). Recent advances in surface engineering and cargo loading techniques have further enhanced the potential of EVs as cancer vaccines by improving antigen loading efficiency, targeting specificity, and immune activation (100, 123). Collectively, these findings underscore the multifaceted role of EVs as a next-generation cancer vaccine platform that integrates antigen delivery, immune stimulation, and adjuvant effects within a single biocompatible vesicle, offering a promising avenue to overcome the limitations of traditional cancer vaccines and improve therapeutic outcomes.

## 5 Preparation, modification techniques, and clinical translation challenges of EV carriers

### 5.1 EVs isolation and purification techniques

EVs isolation and purification are critical steps that significantly influence downstream applications, especially in clinical and therapeutic contexts. Among the most established techniques, ultracentrifugation (UC), density gradient centrifugation (DGC), and immunoaffinity capture remain widely used, each with distinct advantages and limitations. Ultracentrifugation, often considered the gold standard, utilizes high centrifugal forces to sediment EVs based on their size and density. While it enables the processing of large volumes and is relatively accessible, UC is limited by co-isolation of protein aggregates and other contaminants, potential EV aggregation, and lengthy processing times (124, 125). Density gradient centrifugation improves purity by layering samples over density media (e.g., sucrose or iodixanol gradients), allowing EVs to be separated according to their buoyant density. This method achieves higher purity and better separation of EV subpopulations but is more labor-intensive and less scalable for clinical applications (125, 126). Immunoaffinity capture exploits specific surface markers on EVs (e.g., tetraspanins CD9, CD63, CD81) to selectively isolate vesicles using antibody-coated beads or surfaces, offering high specificity and purity. However, this approach is limited by the availability of robust markers, potential bias toward subpopulations, and challenges in releasing intact EVs for functional studies (127, 128).

Beyond these classical methods, emerging technologies such as size exclusion chromatography (SEC), tangential flow filtration (TFF), microfluidics, and charge-based chromatographic techniques have garnered attention for their potential to balance yield, purity, and scalability. SEC separates EVs based on size by passing samples through porous beads, effectively removing smaller protein contaminants and preserving EV integrity. Its gentle nature makes it suitable for sensitive downstream analyses, and it has been successfully applied to isolate EVs from various sources including adipocytes and plasma (129, 130). TFF offers scalable filtration by tangential flow over membranes, minimizing clogging and enabling processing of large volumes with improved purity when combined with other methods like polyethylene glycol (PEG) precipitation and multimodal chromatography (131, 132). Microfluidic platforms integrate multiple isolation principles (size, affinity, electric and acoustic fields) into compact devices, enabling rapid, high-throughput, and potentially point-of-care EV isolation with high purity, although these technologies are still under development for widespread clinical use (133, 134).

Filtration-based methods, including ultrafiltration and novel nanoporous membranes, provide size-based separation and are advantageous for their simplicity and speed. However, they may suffer from sample loss due to membrane fouling or nonspecific binding, and may not fully resolve EV subtypes (135, 136). Charge-based chromatographic methods, such as ion exchange chromatography, exploit the net negative surface charge of EVs to achieve separation. Recent advances in tandem cation and anion exchange chromatography have demonstrated improved purity and yield, particularly for platelet-derived EVs, suggesting that combining charge-based methods with size or affinity techniques could enhance isolation quality (137).

Standardization and optimization of EV isolation protocols are paramount for clinical translation. High purity, high yield, and reproducibility are essential to ensure reliable biomarker discovery and therapeutic efficacy. For instance, combining SEC with ultracentrifugation has been shown to yield EVs with superior purity and functionality suitable for proteomic and RNA analyses (138, 139). Moreover, the choice of isolation method must consider the source material, downstream application, and available resources. For example, bacterial EVs require tailored protocols due to their unique membrane structures, with density gradient centrifugation modes optimized for fecal samples (126, 140). Similarly, plant-derived EVs pose challenges in isolation, with immunoaffinity capture providing precise subclass separation when specific markers are available (127, 141).

In summary, while ultracentrifugation, density gradient centrifugation, and immunoaffinity capture remain foundational techniques for EV isolation, emerging methods such as size exclusion chromatography, tangential flow filtration, microfluidics, and chromatographic charge-based separations offer promising alternatives or complements. Achieving high purity, yield, and standardization is crucial for advancing EVs from bench to bedside, enabling their full potential in diagnostics, therapeutics, and drug delivery. Continuous methodological improvements and consensus on best practices will facilitate reproducibility and clinical applicability of EV-based technologies (124–126, 137, 138).

### 5.2 Functional modification and drug loading strategies of EVs

EVs have emerged as promising natural nanocarriers for advanced drug and vaccine delivery due to their intrinsic biocompatibility, low immunogenicity, and ability to traverse biological barriers. To enhance their therapeutic efficacy and targeting specificity, functional modifications of EVs are indispensable. These modifications primarily focus on two aspects: surface engineering to improve targeting and internal cargo loading to enable multifunctional therapy.

Surface modification of EVs involves decorating their membranes with ligands such as peptides, antibodies, aptamers, or other targeting moieties to improve their homing ability to specific cell types or tissues. This approach addresses the inherent limitations of natural EVs, which often exhibit short circulation times and off-target accumulation, particularly in organs like the liver and spleen. Genetic engineering of donor cells to express fusion proteins, such as CD63 or ARRDC1 fused with therapeutic or targeting proteins, has been demonstrated to increase the loading and surface presentation of desired molecules on EVs, enhancing their tumor-targeting capacity and therapeutic effects (142, 143). Chemical conjugation methods, including click chemistry and avidin-biotin systems, provide versatile and efficient platforms for post-isolation functionalization, enabling stable attachment of ligands without compromising EV integrity (144, 145). Recent advances also include phospholipid-anchored ligand conjugation, which achieves high ligand density and stability on EV surfaces, significantly improving cellular uptake and cytotoxicity in cancer models (146). Importantly, single-vesicle level analyses reveal heterogeneity in ligand conjugation efficiency and highlight the need for precise quantification to optimize targeting performance (147, 148). Surface modifications not only augment targeting but can also reduce immune clearance and improve biodistribution, crucial for clinical translation (149, 150).

Beyond surface engineering, loading EVs with therapeutic payloads such as mRNA, proteins, chemotherapeutic drugs, and nucleic acids enables multifunctional treatment modalities. Various loading strategies have been developed to incorporate cargo into EVs either during biogenesis (endogenous loading) or post-isolation (exogenous loading). Endogenous loading leverages donor cell engineering to package desired molecules into EVs, exemplified by fusion of cargo proteins with EV-enriched proteins like CD63 or TSPAN14, facilitating efficient encapsulation and delivery (151, 152). Exogenous methods include electroporation, sonication, freeze-thaw cycles, and chemical transfection, each with distinct advantages and limitations regarding loading efficiency, EV integrity, and cargo functionality (144, 153, 154). Innovative approaches such as pH-gradient modification enhance nucleic acid loading by transiently acidifying EV interiors, improving encapsulation of negatively charged molecules like miRNA and siRNA without damaging cargo (155, 156). Tonicity control methods have also been shown to increase loading yields for hydrophilic drugs and nucleic acids by reversible membrane permeabilization (157).The cargo spectrum loaded into EVs is broad, encompassing small molecule chemotherapeutics (e.g., doxorubicin, paclitaxel), nucleic acids (mRNA, miRNA, siRNA, CRISPR/Cas9 components), and proteins, including enzymes and transcription factors, enabling gene therapy, immunomodulation, and direct cytotoxicity (158–160). For example, EVs loaded with CRISPR/Cas9 systems have shown promise in gene editing applications, with engineering strategies enhancing packaging and delivery efficiency (159). Additionally, bioengineering EVs to express fusogenic proteins like Syncytin-1 improves cytoplasmic delivery of cargo, overcoming endosomal entrapment (161).To improve loading homogeneity and therapeutic consistency, recent studies employ single-particle resolution techniques to characterize cargo distribution within EV populations, revealing significant heterogeneity that impacts therapeutic outcomes (153, 162). Strategies to isolate and purify lipid-enclosed EVs from contaminants further enhance drug encapsulation quality (162). Moreover, hybrid systems combining EVs with synthetic lipid nanoparticles via microfluidic fusion have been developed to increase drug loading capacity and targeting efficiency (74).

In summary, the functional modification and cargo loading of EVs are critical to unlocking their full potential as multifunctional therapeutic delivery platforms. Surface modifications confer enhanced targeting specificity and biodistribution, while internal loading strategies enable the delivery of diverse therapeutic agents, including nucleic acids, proteins, and chemotherapeutics. Continuous advancements in engineering methods, coupled with precise characterization techniques, are essential to overcome current challenges such as loading heterogeneity, EV yield, and stability. These developments pave the way for the clinical translation of EV-based drug delivery systems with improved efficacy and safety profiles (163–165).

### 5.3 Stability and storage conditions of EVs

The stability and preservation of EVs are critical factors influencing their utility in advanced drug and vaccine delivery systems. EVs, due to their nanoscale size and complex biochemical composition, are susceptible to degradation and functional loss under suboptimal storage conditions. Low-temperature storage, particularly at -80 °C, is widely regarded as the gold standard for long-term preservation of EVs, effectively maintaining their structural integrity, cargo stability, and biological activity over extended periods. Multiple studies have demonstrated that EVs stored at -80 °C retain their morphology, protein content, and nucleic acid cargo with minimal degradation, even after months or years of storage. For instance, Candida albicans-derived EVs preserved at -80 °C maintained both morphological stability and biological function, including protective effects in infection models, whereas repeated freeze-thaw cycles significantly compromised their efficacy (166). Similarly, small EVs (sEVs) stored at -80 °C exhibited superior preservation of particle size distribution, quantity, and cellular uptake compared to storage at higher temperatures (167). However, storage in phosphate-buffered saline (PBS) alone at -80 °C can lead to EV aggregation and loss, highlighting the importance of optimized buffer compositions. The addition of cryoprotectants such as trehalose and albumin to PBS (e.g., PBS-HAT buffer) has been shown to markedly improve EV recovery and stability during freezing and thawing cycles, reducing aggregation and preserving surface proteins critical for cellular interactions (168, 169).

Freeze-drying (lyophilization) emerges as a promising technique to enhance EV stability and facilitate storage at room temperature, which is particularly advantageous for clinical and commercial applications where cold chain logistics pose challenges. Incorporating excipients such as sucrose, poloxamer 188, and novel additives like tryptophan during lyophilization protects EV membranes and cargo, maintaining particle size, morphology, and bioactivity after reconstitution (170, 171). Milk-derived EVs lyophilized with trehalose and tryptophan retained structural integrity and biological function during long-term room temperature storage, representing a significant advancement in EV preservation without reliance on freezing (170). Furthermore, lyophilized EV formulations have been successfully integrated into delivery platforms such as dissolving microneedles, which preserve EV stability and enable controlled transdermal delivery, with maintained activity after months of storage at mild temperatures (172, 173).

Despite these advances, challenges remain in achieving room temperature stability without compromising EV function. Research into stabilizing agents and formulations that protect EVs from oxidative stress, aggregation, and cargo degradation is ongoing. For example, ectoine-enhanced lyophilization has been developed to preserve milk-derived EVs for ocular therapies, demonstrating improved bio-stabilization and therapeutic efficacy after storage at 4 °C (174). Additionally, plant-derived EVs (PDEVs) show promise for oral and systemic delivery due to their inherent stability; however, their preservation benefits from optimized storage conditions and the use of preservatives to maintain size distribution, protein content, and cellular uptake (175, 176). The use of protective excipients such as bovine serum albumin and Tween 20 during storage at 4 °C or -80 °C has been shown to enhance EV recovery and preserve functionality (177).

Moreover, the number of freeze-thaw cycles critically impacts EV stability, often causing aggregation, size increase, and loss of bioactivity. Minimizing such cycles is essential to maintain EV therapeutic potential (169, 178). Alternative approaches, such as accelerated aging protocols involving controlled freeze-thaw cycles, have been proposed to standardize EV preparations and predict long-term storage outcomes (179). The choice of storage container also influences EV stability; low protein-binding tubes reduce vesicle adsorption and loss during storage at 4 °C (180).

In summary, low-temperature freezing at -80 °C combined with optimized buffer formulations currently provides the most reliable means of preserving EV stability for advanced drug and vaccine delivery. Lyophilization with appropriate cryoprotectants offers a promising route to achieve room temperature stability, facilitating transport and clinical application without cold chain dependence. Continued research into stabilizing excipients, storage media, and innovative delivery systems is essential to overcome existing limitations and fully harness the therapeutic potential of EVs in medicine.

### 5.4 Safety and pharmacokinetics in clinical translation

The clinical translation of EVs as advanced drug and vaccine delivery systems necessitates rigorous evaluation of their safety profiles and pharmacokinetic behaviors. A critical aspect is assessing the immunogenicity and toxicity of EVs, which directly impacts their clinical applicability. Mesenchymal stem cell-derived EVs (MSC-EVs), for example, have demonstrated lower immunogenicity compared to their parent cells, reducing risks such as ectopic tumor formation and immune rejection, which are common concerns with cell-based therapies (181, 182). However, despite these advantages, the heterogeneity of EV populations and the source variability pose challenges in ensuring consistent safety outcomes. Immune responses to allogeneic or xenogeneic EVs can lead to immune rejection or adverse reactions, emphasizing the need to address the immunological compatibility of EVs derived from different origins (183). Moreover, the biodistribution and pharmacokinetics of EVs are pivotal to their therapeutic efficacy and safety. Physiologically based pharmacokinetic (PBPK) modeling has emerged as a valuable tool to predict the absorption, distribution, metabolism, and excretion (ADME) of EVs, guiding optimization of EV size, composition, administration routes, and dosing regimens to maximize targeted delivery while minimizing off-target effects (184). For instance, EVs can cross biological barriers such as the blood-brain barrier, enabling delivery to otherwise inaccessible tissues, but this also necessitates careful pharmacokinetic profiling to avoid unintended accumulation or toxicity (181). Clinical studies have begun to reflect these considerations, with early-phase trials focusing on safety endpoints, maximum tolerated doses, and pharmacodynamics, highlighting the importance of standardized manufacturing and characterization protocols to reduce variability and ensure reproducibility (185). Furthermore, the immunogenicity concerns associated with heterologous EVs can be mitigated by engineering strategies such as surface modification or the use of autologous EVs, which may reduce immune clearance and improve therapeutic index (183). In addition, the safety profile must encompass long-term evaluations to detect potential cumulative toxicity or immunosuppression, which remain underexplored in current clinical settings (183). Overall, the translation of EVs into clinical therapeutics demands a comprehensive framework that integrates immunogenicity assessment, toxicity evaluation, and detailed pharmacokinetic studies supported by advanced modeling approaches. Addressing these challenges will be essential to harness the full potential of EVs as safe and effective drug and vaccine delivery platforms in diverse clinical contexts.

### 5.5 Regulatory policies and production standardization

The translation of EVs into advanced drug and vaccine delivery systems necessitates rigorous regulatory frameworks and standardized production protocols to ensure safety, efficacy, and reproducibility. Establishing Good Manufacturing Practice (GMP) compliant production processes is critical for clinical-grade EV manufacturing. This includes defining detailed process requirements encompassing cell source selection, culture conditions, EV isolation, purification, and storage. For instance, the Chinese Society for Stem Cell Research has published the first guideline on the general requirements for producing human stem cell-derived EVs, outlining specifications for preparation, packaging, labeling, and storage to promote institutional protocol acceptance and international standardization (186). Similarly, scalable and reproducible isolation methods such as ultrafiltration combined with size exclusion chromatography (UF/SEC) have been optimized to improve yield and purity of EVs, supporting mass production for clinical applications (187). Quality control systems incorporate characterization techniques following the Minimal Information for Studies of Extracellular Vesicles (MISEV) guidelines, including nanoparticle tracking analysis, Western blotting for EV markers, and electron microscopy, to ensure consistent product identity and purity (188, 189) The development of reference EVs expressing horseradish peroxidase (HRP) further facilitates sensitive quantification and standardization of EV preparations, enhancing quality control during manufacturing and downstream analyses (190).

Regulatory guidance documents are evolving to address the unique challenges posed by EV-based therapeutics. The Japanese Society for Regenerative Medicine issued comprehensive guidance covering risk profiling, manufacturing processes, quality verification, and efficacy assessments to promote high safety standards in clinical EV applications (191). The European Innovation Council’s Extracellular Vesicle Cluster Meeting emphasized the need for application-specific benchmarks, robust manufacturing pipelines, and tailored regulatory frameworks to overcome bottlenecks in EV production and clinical readiness (192). Sterility testing and validation of analytical methods are integral components of quality assurance, with automated sterility testing systems validated for advanced therapy medicinal products including mesenchymal stromal cells and their EVs (193). Moreover, standardization efforts extend to pre-analytical variables and reporting frameworks to enhance reproducibility and reliability of EV research, as exemplified by initiatives like MIBlood-EV for plasma and serum sample quality control (194).

Clinical trial design and regulatory compliance require harmonization of EV isolation, characterization, and functional evaluation protocols. The International Society for Extracellular Vesicles (ISEV) advocates adherence to MISEV guidelines to ensure methodological rigor and comparability across studies (195). Challenges remain in scaling up production while maintaining EV bioactivity and safety profiles, necessitating advances in bioreactor culture systems, purification technologies, and cargo loading methods (196). Regulatory agencies are also addressing the classification of EV-based products, balancing their biological complexity with the need for clear quality and safety standards. The integration of standardized production workflows with regulatory frameworks will be pivotal for advancing EVs from bench to bedside, enabling their potential as next-generation drug delivery vehicles and vaccine platforms (197).

In summary, the establishment of GMP-compliant manufacturing processes, comprehensive quality control systems, and clear regulatory guidelines are essential to realize the clinical potential of extracellular vesicles in advanced drug and vaccine delivery. Ongoing collaborative efforts among researchers, industry stakeholders, and regulatory bodies aim to overcome current challenges in standardization, scalability, and regulatory approval, thereby facilitating the safe and effective translation of EV-based therapeutics into clinical practice (186, 191, 192).

## 6 Conclusion

In conclusion, extracellular vesicles (EVs) are leveraging their unique properties as natural nanoscale carriers to rapidly advance from the forefront of basic biological research to the core stage of biomedical applications. This review, through a systematic analysis, demonstrates that the excellent biocompatibility, inherent targeting and chemotaxis, and the ability to carry diverse biomolecules of EVs make them indispensable in breaking through the bottlenecks of traditional delivery systems (such as excessive immunogenicity and difficulty in crossing physiological barriers), especially in the precise delivery of mRNA vaccines and the combined intervention of cancer immunotherapy, presenting broad prospects.

Although current research on EVs has advanced from the proof-of-concept stage to functional engineering modifications, and has preliminarily verified their safety in multiple clinical trials, compared to the mature industrialization paths of synthetic carriers such as lipid nanoparticles, the EVs field still faces challenges in achieving both high production volume and purity during large-scale production, regulatory standardization issues caused by heterogeneity, and bottlenecks in optimizing drug loading efficiency. However, with the integration of interdisciplinary fields such as microfluidics technology, synthetic biology, and component analysis based on artificial intelligence, these challenges are gradually being overcome.

In the future, the application of EVs will no longer merely focus on simply mimicking synthetic carriers, but will instead place greater emphasis on exploring and leveraging their inherent complexity - for instance, by programming the parent cells to confer them targeted or therapeutic functions, or by fusing the advantages of synthetic materials as “heterogeneous carriers”. It is foreseeable that with the precision of engineering methods and the standardization of production quality control, EVs are expected to transform from a “potential tool” into a next-generation drug and vaccine delivery platform that truly addresses clinical needs and drives therapeutic innovation.
